# Neurophysiological Characterization of a Non-Human Primate Model of Traumatic Spinal Cord Injury Utilizing Fine-Wire EMG Electrodes

**DOI:** 10.3390/s19153303

**Published:** 2019-07-27

**Authors:** Farah Masood, Hussein A. Abdullah, Nitin Seth, Heather Simmons, Kevin Brunner, Ervin Sejdic, Dane R. Schalk, William A. Graham, Amber F. Hoggatt, Douglas L. Rosene, John B. Sledge, Shanker Nesathurai

**Affiliations:** 1 School of Engineering, University of Guelph, Guelph, ON N1G 2W1, Canada; 2 The Department of Biomedical Engineering, Al-Khwarizmi College of Engineering, Baghdad University, Baghdad 47146, Iraq; 3 The Wisconsin National Primate Research Center, University of Wisconsin-Madison, Madison, WI 53715, USA; 4 The Swanson School of Engineering, University of Pittsburgh, Pittsburgh, PA 15261, USA; 5 The Division of Physical Medicine and Rehabilitation, Department of Medicine, McMaster University, Hamilton, ON L8S 4K1, Canada; 6 The Center of Comparative Medicine, Brigham and Women’s Hospital, Boston, MA 02115, USA; 7 The Department of Anatomy and Neurobiology, Boston University School of Medicine, Boston, MA 02118, USA; 8 The Lafayette Bone and Joint Clinic, Lafayette, LA 70508, USA; 9 The Department of Physical Medicine and Rehabilitation, Hamilton Health Sciences, St Joseph’s Hamilton Healthcare, Hamilton, ON L9C 0E3, Canada

**Keywords:** fine-wire intramuscular EMG electrode, non-human primate model, traumatic spinal cord injury, wavelet transform, relative power, linear mixed model

## Abstract

This study aims to characterize traumatic spinal cord injury (TSCI) neurophysiologically using an intramuscular fine-wire electromyography (EMG) electrode pair. EMG data were collected from an agonist-antagonist pair of tail muscles of Macaca fasicularis, pre- and post-lesion, and for a treatment and control group. The EMG signals were decomposed into multi-resolution subsets using wavelet transforms (WT), then the relative power (RP) was calculated for each individual reconstructed EMG sub-band. Linear mixed models were developed to test three hypotheses: (i) asymmetrical volitional activity of left and right side tail muscles (ii) the effect of the experimental TSCI on the frequency content of the EMG signal, (iii) and the effect of an experimental treatment. The results from the electrode pair data suggested that there is asymmetry in the EMG response of the left and right side muscles (*p*-value < 0.001). This is consistent with the construct of limb dominance. The results also suggest that the lesion resulted in clear changes in the EMG frequency distribution in the post-lesion period with a significant increment in the low-frequency sub-bands (D4, D6, and A6) of the left and right side, also a significant reduction in the high-frequency sub-bands (D1 and D2) of the right side (*p*-value < 0.001). The preliminary results suggest that using the *RP* of the EMG data, the fine-wire intramuscular EMG electrode pair are a suitable method of monitoring and measuring treatment effects of experimental treatments for spinal cord injury (SCI).

## 1. Introduction

Traumatic spinal cord injury (TSCI) is a serious neurological condition. Worldwide, there are approximately 180,000 new cases each year [1,2]. The most common causes of TSCI are motor vehicle collisions, falls, sports-related activities, and interpersonal violence [2,3]. TSCI typically damages the motor, sensory and autonomic fibre tracts. Patients experience a spectrum of clinical abnormalities such as limb paralysis, dysesthesia, as well as bowel and bladder dysfunction [3,4,5]. The burden of this condition is shared by the person affected, family members, the community, as well as the healthcare system. Long-term survival and quality of life have improved due to enhanced rehabilitation and medical interventions. However, it has not been convincingly demonstrated that current pharmacological treatments have substantially improved spinal cord function.

Conceptually, the understanding of TSCI impairments and recovery can be advanced through animal models [4,5,6,7,8,9]. Non-human primate models (NHP) are particularly valuable due to the anatomic and physiological similarities between NHP and human beings [6,7]. The tail in NHPs is analogous to a human limb. The tail is integral to performing functional tasks such as standing on hindlimbs as well as reciprocal movements that aid in balance during ambulation [10]. As described later in this paper, the tail exhibits other features analogous to human beings such as limb dominance or preference. 

SCI impairments are usually measured by observing behaviours as well as histopathological assessment [11]. Electromyography (EMG) signals confer certain advantages over these other methods. It is particularly useful for assessing TSCI as EMG data collected on a serial basis as this approach facilitates the characterization of motor unit (MU) activation and recruitment [8]. EMG signals can be recorded through multiple channels, permitting the simultaneous assessment of multiple muscle groups. This is particularly helpful in evaluating agonist–antagonist muscle pairs, in which the assessment of muscle co-contractions are associated with central nervous system disorders [4,9]. 

EMG data can be obtained through surface recording or intramuscular electrodes placed into the muscles. There is limited literature regarding the nature of intramuscular EMG signals after TSCI in animals and humans. The majority of human studies have been based on surface EMG electrodes. As a general construct, TSCI results in a perturbation of the EMG signal. Calancie et al. [12] studied the recovery of volitional activity after acute SCI in human beings utilizing pairs of surface EMG electrodes. This study demonstrated perturbations in some EMG characteristics including abnormalities in recruitment. Lewko [13] utilized surface EMG electrodes and noted disturbed behaviour in spinal cord conductivity with quiet standing. Nout and Rosenzweig among others [8], ref. [9] developed a NHP model of SCI, and the results demonstrated a significant difference in EMG amplitude and temporal patterns between the healthy and the SCI subjects. Also, they noted uncoordinated muscle activity during the post-lesion condition. Wiegner et al. and Shahani et al. studied the recruitment pattern of MUs post-SCI [14] and cortical pathology [15] using needle EMG signals. The results indicate that MUs fire irregularly with low discharge rate post-SCI. Also, the inter-discharge intervals (IDIs) have a positive serial correlation which results from the decreased variability in length of the adjacent intervals. Capogrosso et al. [16] developed a brain-spine interface to modulate the consequences of TSCI in NHP. EMG signals during continuous locomotion were recorded and averaged to calculate the spatiotemporal maps of the motoneuron activation in monkeys. Their results suggested a practical translational pathway for conceptual analysis studies and investigational applications in human with SCI.

The bio-signals such as EMG are non-stationary signals. Furthermore, EMG signals during volitional muscle contraction have a random nature which means the active MUs have an irregular firing rate [17]. Thus, determining the method to select relevant features of the EMG signal becomes challenging. In this context, several signal processing techniques have been tested, with the goal of developing a robust method [18]. To advance this goal, mathematical transformations can be utilized. Specifically, the EMG signal could be represented in different domains; time, frequency, or a time-frequency/wavelet domain. The amplitude and frequency content of the EMG signal help in understanding the physiology and the pathology aspects of muscle activity. The frequency content of the EMG signal mainly has been calculated using the fast Fourier transform (FFT). However, FFT may not be an appropriate choice for some cases such as the dynamic and variant level of contractions [19,20]. To overcome this problem, the wavelet transforms (WT) is applied. The WT is an efficient mathematical analysis method for temporally nonstationary and spatially nonhomogeneous bio-signals [21]. Also, this approach has proved its ability to represent precise measurements and to extract useful information from non-stationary biomedical signals [22,23,24,25]. Decomposition, denoising, and pattern classification are the most common applications of the WT in the EMG field [18,22,26,27,28,29,30,31,32,33,34]. Phinyomark et al. [30] investigated the usefulness of extracting EMG features from the multi-resolution wavelet decomposition process. The results showed that the reconstructed EMG of the first and second level (detail coefficients) have improved the class separability. Yamada et al. [26] introduced a new EMG decomposition algorithm by adopting the principal component analysis of wavelet coefficients. This method showed a higher decomposition accuracy when compared to conventional wavelet methods. Fang et al. [27] decomposed EMG signals into their constituent single motor units using wavelet spectrum matching, and the results were satisfying. 

The intramuscular fine-wire electrode data of this experiment is particularly unique as it is collected both prior and subsequent to a TSCI. The longitudinal nature of the data, however, presents the challenge of limiting the sampling rate of the sensors to allow for long-term monitoring. In addition, free and dynamic movements must be permitted to allow for data collection that reflected the subjects true volitional control as the limb would be naturally used. Thus, conventional decomposition methods of EMG data are not suitable for this problem as these methods rely on higher sampling frequency (around 25 kHz) [35] as well as the need for isometric contractions [36,37,38,39]. 

In this work, the intramuscular fine-wire EMG data have been analyzed using the WT as an EMG decomposition method and relative power (RP) as a metric of the active MUs within each WT sub-band. Specifically, this work will address the following questions:

1. Is the intramuscular fine-wire electrode pair data capable of detecting limb dominance in the subjects prior to lesion?
2. In the post-lesion period, is there a change in EMG activity attributed to the experimental spinal cord injury and how it could be characterized in term of frequency content?
3. What is the difference in the EMG activity between the control and the treatment group in the post-lesion period (i.e., is there a treatment effect)?

The collected intramuscular EMG dataset contains information obtained before and after an experimental TSCI, allowing each subject to serve as normal control. Accurate measurement of impairment and recovery in a model of TSCI has significant implications for the identification and development of TSCI therapeutics. With no standardized model for combined pre-lesion, post-lesion, and recovery analysis, WT with multi-resolution data could provide evidence of being able to account for multiple effects in a single model. The extended recording period both before and after TSCI in the NHP model is necessary to replicate TSCI; recovery in humans will be recognized months after injury. Repeated measurements in the NHP model permit the long-term evaluation effects of experimental therapies. It is expected that the developed NHP model and its preliminary results will provide a better understanding of the TSCI and may help with the prediction of recovery in human limbs.

## 2. Materials and Methods

The focus of the SCI model was to create a lesion that mimics human SCI without clinical impairments. The experimental methods to create the lesion are described in detail in previous publications [4,7], but are presented here briefly. The subjects, six adult Macaca fasicularis monkeys, underwent two surgical procedures. The first to insert a small transmitter (PhysioTel D70, Data Sciences International, St. Paul, MN, USA) with attached fine-wire intramuscular electrode pair into the lower back of the subjects. The electrodes were implanted into the left and right flexor cauda longus and brevis muscles (tail muscles). The EMG signals were measured using the fine-wire electrodes with 10 mm inter-electrode distance, 10 mm exposed wire length, 1000 Hz sampling frequency, and common ground. 

**Figure 1:**
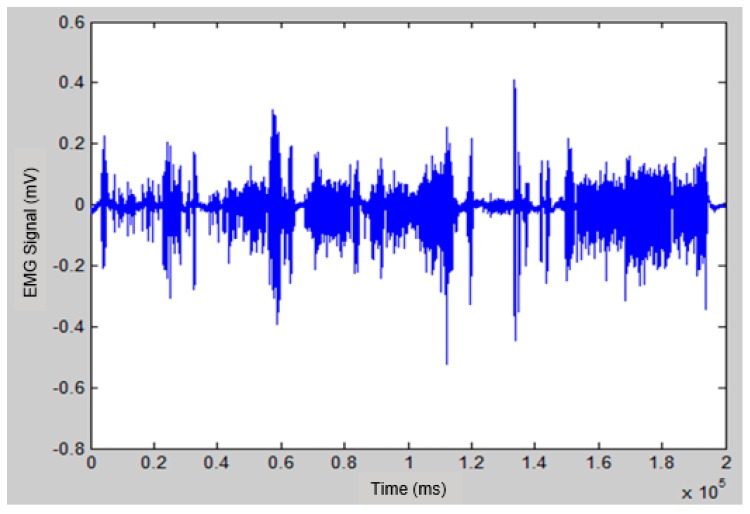
Graphical representation of the raw electromyography (EMG) signal.

Baseline pre-lesion EMG data from the fine-wire electrode pair were collected during voluntary movement of NHPs within their home enclosures for 30 days. The data were collected from Monday to Friday (excluding holidays) for approximately 1 h per day. After 30 days, the subjects underwent a second surgical procedure. A small laminotomy was completed at the L5 vertebral level. An epidural balloon catheter was inserted and advanced approximately 10 cm cranial to the level of the lower thoracic spinal cord. The lesion was created by inflation of the balloon with air. The balloon remained inflated for 60 s and then was deflated. The catheter was then removed, and the surgical incision closed. The lesions initially disrupted the grey and white matter at the site of lesion creation. The histopathological features, that were created by this method are similar to human TSCI [4]. To mimic the time frame between human injury and administration of emergency medical treatment, the subjects remained under anesthesia for one hour. Four subjects that received the experimental combination treatment for 90 days while two subjects did not receive any treatment. Combination treatment consisted of a bolus 0.2 mg/kg bolus of thyrotropin releasing hormone (TRH), followed by a continuous infusion of 0.2 mg/kg per hour for 1 h. These subjects were also treated with 60 mg of selenium and 80 IU of vitamin E daily. TRH is a tripeptide produced by the hypothalamus; selenium and Vitamin E are antioxidants. The combination of these agents may modulate the physiological sequelae of TSCI [40]. This protocol was approved by the Institutional Animal Care and Use Committee (IACUC) at Harvard University and the University of Wisconsin at Madison. As such, EMG data from the tail is analogous to EMG data obtained from the limb of a human being with TSCI EMG data were collected from all six subjects. However, in one subject, the data from one side was not recorded due to a technical error. As such, raw data in this paper represents the experience of three subjects that received the combination therapy, and two subjects who did not receive treatment. Figure 1 illustrates a sample of the recorded raw EMG data.

**Figure 2:**
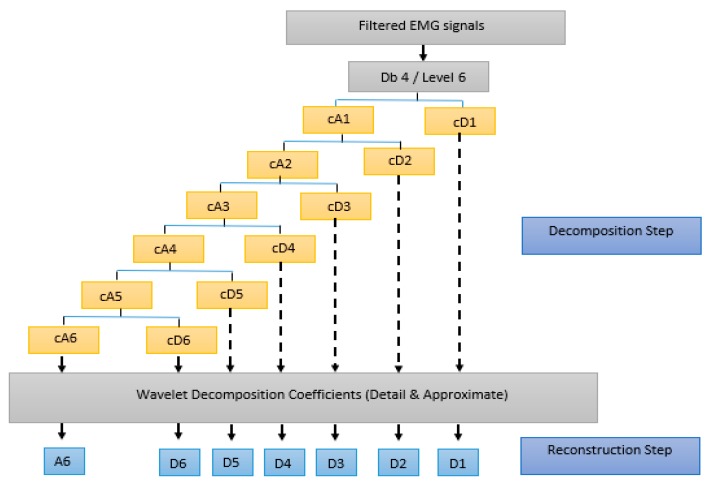
Wavelet analysis; decomposition and reconstruction steps.

In this NHP model, the induced lesion produced a TSCI limited to the upper motor neuron tracts that supply the lower motor neurons of the tail muscles [7]. Lesion of such nature result in a perturbation in the MUs discharge properties; i.e., abnormalities in the inter-discharge interval, firing rate, and floating serial correlation coefficient [14,41]. These abnormalities have been typically characterized in non-physiological experimental conditions such as low force levels and isometric contraction frequency [14,37,38,39]. In this work, the EMG data were collected when the subjects engage in physiological activities (i.e., locomotion in a cage). The proposed EMG analysis method consists of the following three steps:

- The raw EMG data obtained from daily recordings were filtered using a bandpass filter (4th order Butterworth filter with a lower and an upper cut off frequency of 10 and 450 Hz respectively). A notch filter with 60 Hz was also applied to eliminate the power line noise, and the input signal was processed forward and backward to solve phase shift problems. The EMG conditioning steps have been implemented using MATLAB software (MathWorks, Natick, MA, USA).
- A decomposition process was applied using wavelet transforms. Each WT sub-band was assumed to represent the firing rate of a group of MUs. Also, it was assumed that the *RP* of each individual sub-band reflects the level of activity for these MUs. Thus, increases or decreases in *RP* may characterize the recruitment pattern process of the MUs through different conditions of the experiment. The filtered EMG signals were broken down into seven frequency sub-bands using the WT. The discrete wavelet transforms (DWT) was selected for this work because it has non-redundant results, and it required less computational time and costs [42,43]. A Daubechies mother wavelet of fourth order ‘db4′ was used due to its similarity to the triphasic pattern of the motor unit action potential [44]. Consistent with the analysis of other bio-signals, DWT decomposition was performed using six levels [43,45,46,47,48]. The wavelet analysis was performed in two steps, as presented in Figure 2:
  1. The EMG signals were decomposed into seven sub-bands, one approximate coefficient (cA6), and six detail coefficients (cD1, …, cD6).
  2. The EMG signal was then reconstructed at each level using inverse discrete wavelet transform, and seven EMG reconstructed signals (A6, D1, …, D6) were obtained from their coefficients (cA6, cD1, …, cD1). Table 1 shows the frequency ranges of the seven EMG sub-bands.
- To evaluate the changes in EMG sub-bands during different phases of the experiment, these changes were characterized using the RP. The probabilistic distribution of the spectral power was quantified by calculating the relative power of each spectral component [49]. To obtain the RP, firstly the power spectral density was determined for each reconstructed EMG sub-band signal. Then, the *RP* for each individual sub-band was calculated using the following formula [50]:
  (1) $$RP\left(\%\right)=\frac{SBP}{TP},$$
  where:
  *RP:* the relative power of the desired sub-band.
  *SBP:* the power of the desired sub-band (e.g., A6, D1, … or D6).
  *TP:* the total power of all the sub-bands (A6 + D1, …, + D6).

**Figure 3:**
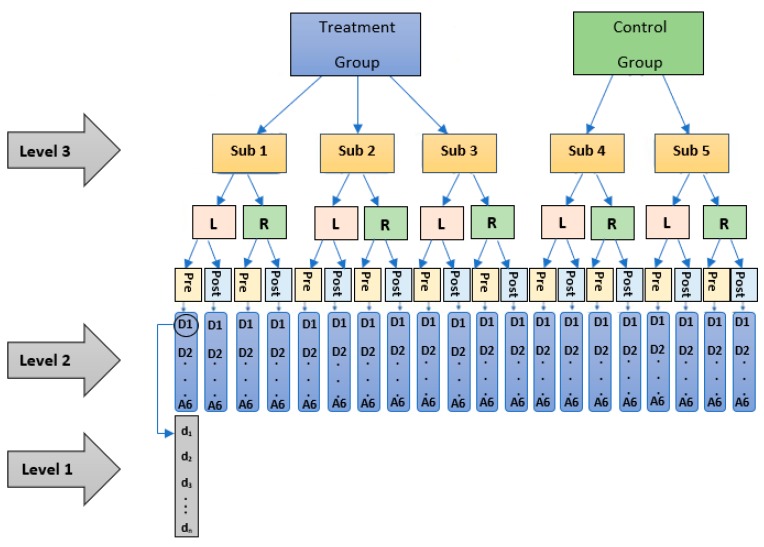
Data Structure: The data in this work was collected from the left (L) and right (R) side of the tail for five subjects during multiple experiment days (d1, d2, …, dn) for (pre- and post-lesion period). The data of each day was decomposed into seven frequency sub-bands (D1, D2, D3, …, A6). Three of the subjects received a combination of treatment (Treatment group); the remaining two subjects did not receive any treatment post-lesion (Control group).

The goal was to address the research questions by analyzing specific subsections of the data and testing specific effects. To test the lateralization effect (attributed to limb dominance), only the pre-lesion data were considered for the two sides (left and right). Then, the lesion effect was tested using the data from both pre-and post-lesion periods, and two separate models were fitted one for each side. To test the treatment effect, two separate models (left and right) were fitted using only the post-lesion data. The data in this work is considered a clustered longitudinal dataset with three levels; days are nested in frequency sub-bands, which are in turn nested within subjects (day: is level 1, sub-bands: is level 2, and subject: is level 3). As a result of such hierarchical structure as in Figure 3, the non-independence problem of the observations would arise which requires an appropriate statistical analysis method. A mixed model is a statistical method that was developed particularly to address the non-independence (correlated, or repeated measurements) issue by including the random effect term in its model. The developed mixed models controlled for the within-subject correlation by including a random effect for the subject and the nested sub-bands within each subject, with a variance-covariance structure and restricted maximum likelihood estimation. The models were implemented in the statistical software R using the (lme4) package. The significance level was set to (α=0.05). The *RP* for any given subject at (day) *i* for (sub-band) *j* nested within (subject) *k* denoted as *RPijk*, is represented in the following equation (see a summary of parameters in Table 2):

(2) $$\begin{array}{l} RP_{ijk}=\beta_{0}+\beta_{1}\;Day_{ijk}+\beta_{2}\;EFFECT_{jk}+\beta_{3}\;EFFECT_{jk}\;Day_{ijk}+\;\beta_{4}Freq_{jk}+\\ \beta_{5}\;Freq_{jk}\;Day_{ijk}+\beta_{6}\;Freq_{jk}\;EFFECT_{jk}+\;\beta_{7}\;Freq_{jk}\;EFFECT_{jk}\;Day_{ijk}\;\left(fixed\right)+\\ \alpha_{0k}+\;\alpha_{1k}\;Day_{ijk}+\alpha_{0jk}+\alpha_{1jk}\;Day_{ijk}+\varepsilon_{ijk}\;\left(random\right), \end{array}$$

## 3. Results

The results were presented in the form of answers to the three research questions that were stated in the introduction. They are as follows:

*(1)*  Is the intramuscular fine-wire electrode pair capable of detecting limb dominance in the subjects prior to lesion?

**Figure 4:**
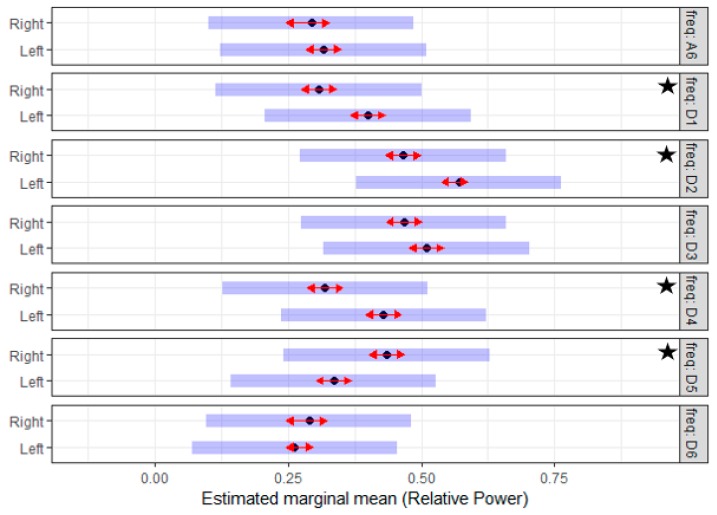
The estimated mean of the relative power (*RP*) of seven reconstructed EMG sub-bands prior to the creation of traumatic spinal cord injury (TSCI) for the left and right side of the tail. Of note for each band, there is a difference in *RP* value when compared to the left and right side of the tail. The D2 sub-band of the left and right side has the maximum RP, and the significant difference between the two sides is at the D1, D2, D4, and D5 sub-bands. The star indicates a significant difference.

To test the symmetry of tail muscle activity on both sides, the *RP* of the EMG signals during the *pre-lesion* period were analyzed using a linear mixed model. The interaction of the frequency sub-band and the side variable in the statistical model demonstrated a significant effect (*p*-value < 0.001). Given that the (side*frequency) interaction is significant, Tukey’s mean comparisons were generated to identify what means differ significantly. These results suggested that side variable had a significant effect on the *RP* value of the D1, D2, D4, and D5 sub-bands. The estimated mean of the *RP* for the D1, D2 and D4 sub-bands of the *left side* were significantly higher than that of the *right side.* On the contrary, the estimated mean of the *RP* for the D5 sub-band on the *left side* was significantly lower than that of the *right side.* The estimated mean for the *RP* across the different frequency sub-bands for both sides is illustrated in Figure 4. Taken together, these results showed that the left and right tail muscles have asymmetry activation.

*(2)*  *In the post-lesion period, is there a change in the EMG activity attributed to the experimental spinal cord injury and how it could be characterized in terms of RP?*

**Figure 5:**
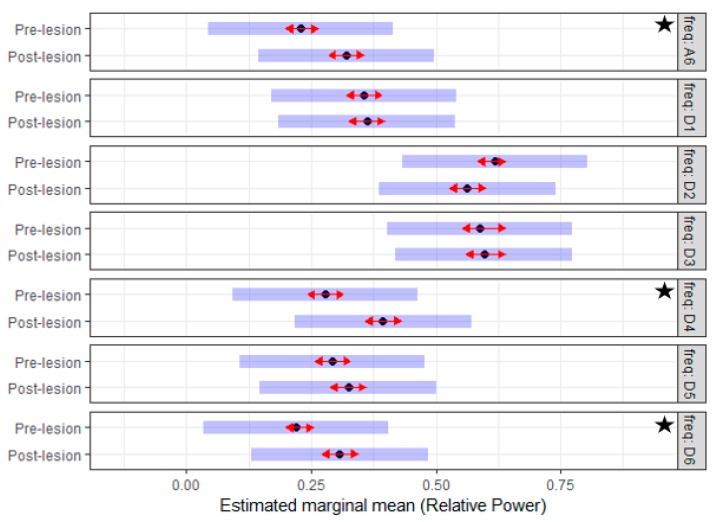
The estimated mean of the *RP* of seven reconstructed EMG sub-bands prior and post to the creation of TSCI for the *left side* of the tail. Of note, the *RP* values for the frequency sub-bands (D4, D6, and A6) are significantly higher in the post-lesion period. The star indicates a significant difference.

**Figure 6:**
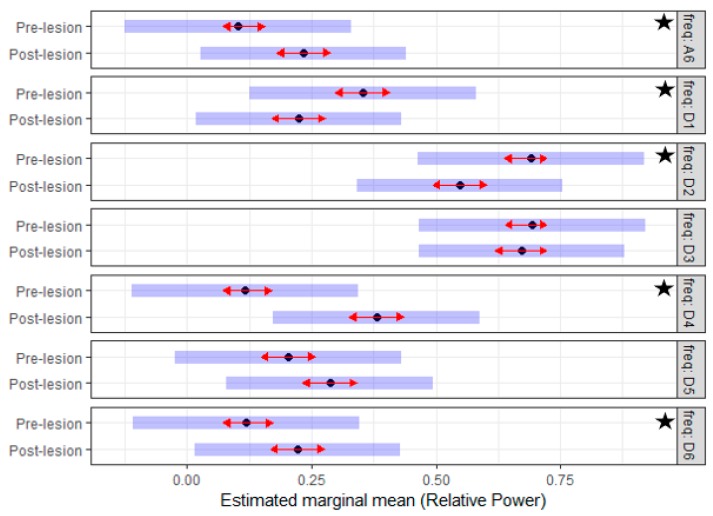
The estimated mean of the *RP* of seven reconstructed EMG sub-bands prior and post the creation of TSCI for the *right side* of the tail. Of note, the *RP* values for the lower frequency sub-bands (D4, D6, and A6) are significantly higher in the post-lesion period, while the higher frequency sub-bands (D1 and D2) are significantly lower in the post-lesion period. The star indicates a significant difference.

To answer this question, the effect of the created lesion was analyzed using a linear mixed model. The lesion effect was studied by testing the difference between the EMG characteristics during the *pre-* and *post-lesion* period. The interaction of the frequency sub-band and the lesion variable in the statistical models of both sides demonstrated a significant effect (*p*-value < 0.001). Given that the (lesion*frequency) interaction was significant, Tuckey’s means comparisons were generated to identify which means differ significantly. Figure 5 and Figure 6 summarized the estimated mean of the *RP* values for different frequency sub-bands of the *pre-* and *post-lesion* group for the two sides. On the *left side*, the estimated mean of the *RP* for the D4, D6 and A6 sub-bands was significantly higher in the *post-lesion* period compared to the *pre-lesion* period. On the *right side*, the estimated mean of the *RP* for the D4, D6, and A6 sub-band was also higher significantly in the *post-lesion* period. On the other hand, the estimated mean of the *RP* for the D1 and D2 sub-bands was significantly lower in the *post-lesion* period compared to *pre-lesion* period. The results suggested that the created lesion had a clear effect on the discharge properties of MUs, and with this technique, changes in discharge properties can be detected even when there is no clinical evidence.

*(3)*  *What is the difference in the EMG activity between the control and the treatment group in the post-lesion period (Treatment effect)?*

**Figure 7:**
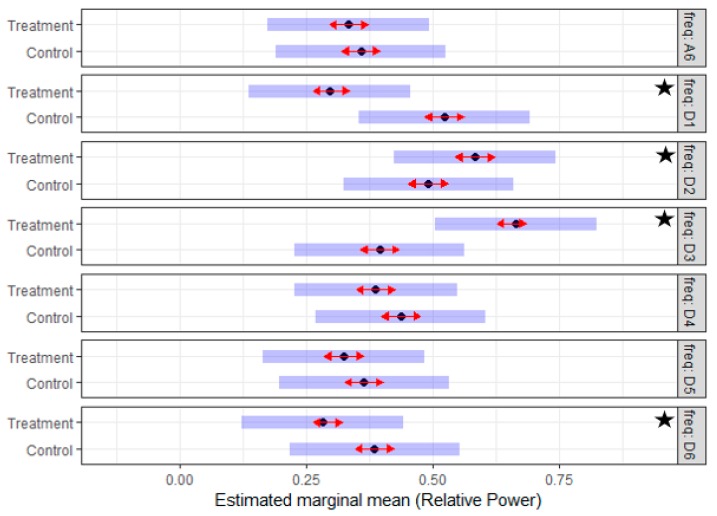
The estimated mean of the *RP* of seven reconstructed EMG sub-bands post-lesion for the treatment (Tr) and the control (Ctrl) groups of the *left side*; of note, the *RP* values for the frequency sub-bands (D1, D2, D3, and D6) are significantly different. Subjectively, the distribution of the *RP* in the treatment group is similar to the *RP* distribution in the pre-lesion period for all the subjects. The star indicates a significant difference.

**Figure 8:**
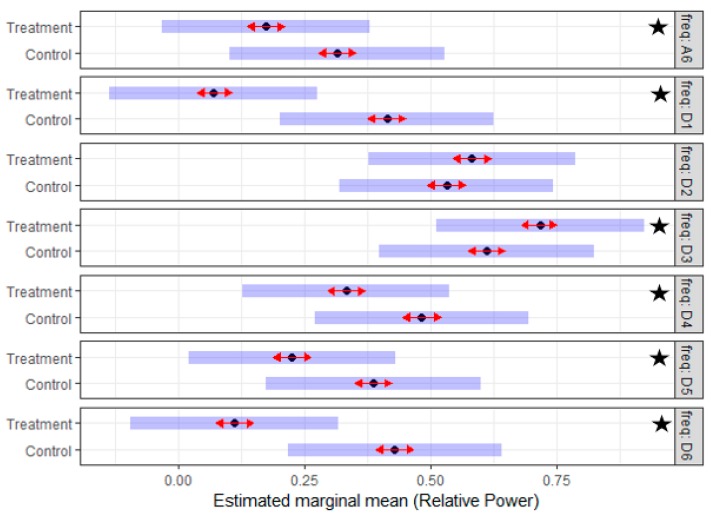
The estimated mean of the *RP* of seven reconstructed EMG sub-bands post-lesion for the treatment (Tr) and the control (Ctrl) group of the *right side*; of note, the *RP* values for frequency sub-bands (D1, D3, D4, D5, D6, and A6) are significantly different. Subjectively, the distribution of the *RP* in the treatment group is similar to the *RP* distribution in the pre-lesion period for all the subjects. The star indicates a significant difference.

The *post-lesion data* were utilized to generate two separate mixed models, one for each side. The potential effect of the treatment was analyzed using the *RP* of the left and the right sides incorporating an analysis which compared the treatment and the control groups. The interaction of frequency sub-band and treatment variables in the models of both sides demonstrated a significant effect (*p*-value < 0.001). Given that the (treatment*frequency) interaction was significant, Tukey’s mean comparisons were generated to identify what means differ significantly. Figure 7 and Figure 8 summarize the estimated mean of the *RP* values for different sub-bands of the control and treatment group for the two sides. On the *left side*, the D1, D2, D3, and D6 sub-bands have a significant difference, while on the *right side* the effect was significant in all the sub-bands except the D2. The results suggested that there is a significant difference in the discharge properties of MUs of the treatment and the control groups during the post-lesion period.

## 4. Discussion

### 4.1. Non-Human Primates Appear to Exhibit Limb Dominance

In human beings, the construct of limb dominance or preference is well accepted; i.e., most people use their right arm for functional tasks, and a minority use the left arm. A select few people can use both arms equally well (ambidextrous). The question of whether monkeys have limb dominance remains a subject of scientific inquiry. In this analysis, there is asymmetry related to the *RP* of EMG data derived from the left and the right tail muscles. These results suggest that the long-tailed macaque (*Macaca fascicularis*) exhibits limb preference and/or dominance. This is also consistent with the observations of the veterinary staff involved with this project. Generally, limb dominance refers to the preferential use of one limb to perform functional tasks [51]. This asymmetry in the pre-lesion (normal control) period has implications for any analysis subsequent to the experimental TSCI. Conceptually, volitional motor control involves a two-circuit pathway; i.e., the upper motor neurons and the lower motor neurons of the corticospinal tracts. The upper motor neurons originate in the cortex, travel the internal capsule and pyramids, and terminate into the grey matter of the spinal cord. The lower motor neurons originate in the grey matter of the spinal cord, exit via the nerve roots and reach the muscles via the plexus and peripheral nerves. The data suggest that the neural network that controls the left and right neurophysiological circuit is not symmetrical. Therefore, it stands to reason that experimental TSCI will have different effects on the separate circuits. This is consistent with human disease, to the extent that a lesion in one part of the central nervous system may have different manifestations related to the limbs. As such, the effects of the lesion, from a neurological perspective, were analyzed in model 2 separately for both the left and the right side.

### 4.2. Experimental Traumatic Spinal Cord Injury (TSCI) Causes Perturbation of Electromyographic (EMG) Data

The experimental TSCI resulted in both a physiological and histopathological perturbation [4]. In this analysis, there was a difference in the *RP* values when comparing the pre- and post-lesion data; this was statistically significant. Specifically, the low frequency (LF) sub-bands (D4, D6, and A6) were increased significantly during the post-lesion period on both sides. Also, the high frequency (HF) sub-bands (D1 and D2) were decreased significantly during the post-lesion period on the right side. These results are consistent with the literature [14]. As a general construct, neurological disease is associated with abnormalities in the firing of the motor units and leads to a lower discharge frequency [14,15,52]. This, in turn, can affect the distribution of the high and low-frequency components of EMG signals. This shift in the *RP* value may be related to the disorganized, spontaneous firing of single or multiple motor units, which affect the frequency distribution. Speculatively, a component of these abnormalities was be related to spasticity; in TSCI, there may preferentially affect the recruitment of Type I or Type II motor units, which could also distort the frequency content. The elucidation of these relationships requires further research. With this method, the change in recruitment behaviour post the TSCI might be detectable even before the clinical evidence. 

### 4.3. Combination Treatment Is Associated with Treatment Effect

The combination of TRH, selenium and vitamin E is associated with a treatment effect. Specifically, three subjects received a combination treatment and two subjects did not receive treatment. In the post-lesion period, there was a significant difference in *RP* values for most of the frequency sub-bands of the treatment and the control groups. These results suggested there is a clear difference in the discharge properties of MUs of the treatment group during the post-lesion period. This finding was present for both the left and the right side. However, in the context of a limited number of subjects, this should be considered as a preliminary inference. The rationale for this combination treatment in TSCI and the implications thereof are published elsewhere [40].

### 4.4. Recording of EMG Signals from Surface, Needle and Wire Electrodes

There are a variety of methods used to obtain EMG data from humans and animals. As a general construct, there are three types of electrodes: surface, needle, and wire electrode. The neurophysiological characteristics and the interpretation of the data derived are not synonymous. Surface electrodes are placed over the skin; they can be manufactured from a variety of materials and formed into various sizes and shapes. Surface recording over the skin is subject to distortion of the EMG signal due to the interposition of the fascia and the subcutaneous tissues. With limb movement, the skin, the subcutaneous tissue and the muscles move asymmetrically. This contributes further to movement artifact. These tissues are effectively a low pass filter. In comparison, needle electrodes are made of metal; subtypes include monopolar, concentric, single fibre and macroelectrodes. Wire electrodes, in contrast, can vary in diameter and length. Wire electrodes allow for long term implantation in subjects and are generally well tolerated. When compared to surface electrodes, there is less cross-talk. As a conceptual argument, data from wire electrodes aggregates the EMG signal from muscle fibres in multiple motor units. The signal is potentially affected by multiple factors including the proximity of the signal generator to the wire, phase cancellation, size of fibres, and type of fibres (i.e., fast twitch, slow twitch). As well, there is some contribution related to the neural network that controls the agonist–antagonist pair. 

The fine-wire recordings from this model are particularly unique. Specifically, the EMG data were obtained prior and subsequent to TSCI. Additionally, the data were obtained while the subjects were moving freely (dynamic movements), over an extended period of time. Some potential limitations include the relatively few numbers of subjects; this is, in part related to cost. As well, the collection of longitudinal nature precluded a very high sampling rate. 

## 5. Conclusions

In conclusion, this research characterizes the frequency content of intramuscular EMG signals recorded pre- and post-TSCI in a validated NHP model. The EMG data were collected in a longitudinal nature and were obtained from the left- and right side of the Macaca fasicularis tail muscles engaged in the dynamic/free activity. In the absence of the TSCI, the EMG data demonstrated an asymmetry activation of the two sides; this is consistent with the human phenomenon of limb preference and/or dominance. Perturbation with an experimental lesion resulted in a clear EMG consequence. Of note, the effect of the lesion was different on the left and the right side. Also, there is a preliminary inference that treatment with a combination of TRH, selenium and vitamin E may improve recovery. The results indicate that the *RP* of the decomposed EMG data adds a new dimension to the evaluation of impairment and recovery in this NHP model of experimental TSCI. This analysis might lead to a new assessment index for motor unit activity and progression of recovery from TSCI.

## Figures and Tables

**Table 1:** The frequency ranges of the seven EMG reconstructed sub-bands.

Wavelet Decomposition Level	Frequency Range/Hz	Reconstructed EMG Sub-Bands
1	250–500	D1
2	125–250	D2
3	62.5–125	D3
4	31.25–62.5	D4
5	15.63–31.25	D5
6	7.81–15.63	D6
6	0–7.81	A6

**Table 2:** Parameters used in Equation (2).

Parameter	Definition
$RP_{ijk}$	response (relative power)
$Day_{ijk}$	experiment day
$Freq_{jk}$	frequency sub-band (D1, D2, D3, D4, D4, D6, and A6)
**EFFECT**	the main effect in the models, so for:
	Model 1: the side effect (categorical variable with two levels (left and right side))
	Model 2: the lesion effect (categorical variable with two levels (pre- and post-lesion))
	Model 3: the treatment effect (categorical variable with two levels (control and treatment group))
$\beta_{0}\;through\;to\;\beta_{7}$	the fixed effect associated with the (intercept, Day, EFFECT, EFFECT*Day, Freq, Freq*Day, Freq* EFFECT, and Freq* EFFECT*Day)
$\alpha_{0k}$ $,\;\alpha_{1k}$	the subject random effect associated with the intercept and Day slope, respectively
$\alpha_{0jk}$ $,\;\alpha_{1jk}$	the random effect of a frequency sub-band nested within a subject associated with the intercept and Day slope respectively
$\varepsilon_{ijk}$	random error
